# Comparison of mobile health education messages verses face-to-face consultation for weight reduction among overweight female adolescents in Thailand

**DOI:** 10.12688/f1000research.51156.1

**Published:** 2021-07-22

**Authors:** Supim Wongtongtair, Sompoch Iamsupasit, Ratana Somrongthong, Ramesh Kumar, Khemika Yamarat

**Affiliations:** 1 Health Promotion, Srinakharinwirot University, Ongkharak, Nakhon-Nayok, 26120, Thailand; 2 Collage of Public Health Science, Chulalongkorn University, Prathumwan, Bangkok, 10330, Thailand; 3 Health Services Academy, Health Services Academy, Islamabad, Islamabad, Pakistan

**Keywords:** Electronic health education, Facebook, health education, health belief model, social support, obesity, Thailand, health behaviors

## Abstract

**Background**: Obesity is considered a significant public health problem in Thailand. This study was conducted to compare the impact of mobile health education messages verses face-to-face consultation on weight reduction among overweight female university students.

**Methods**: This comparative cross-sectional study comprised three groups: a control group, a group receiving mobile health education, and a group receiving face-to-face consultation. Each group contained 26 participants taking part over a period of 12 weeks, with a 12-week follow-up thereafter. The data analysis used two-way repeated measures ANOVA with least significant difference testing. The study was ethically approved at Chulalongkorn University, Thailand.

**Results**: The results revealed that the group receiving mobile health education had the lowest average body mass index and waist–hip ratio after intervention ( *p* < 0.05). In addition, both intervention groups significantly improved their health belief, social support, and health behavior scores in comparison to the control group ( *p* < 0.001). The results show that the average scores for social support for eating and exercise at baseline were significantly lower than at post-intervention or follow-up ( *p* < 0.001). In addition, the results of both aspects of social support showed that the average social support score at post-intervention was significantly higher than at follow-up. Furthermore, the health behavior score measured post-intervention was higher than at follow-up. There was a statistically significant difference in average metabolism during physical activity ( *p* < 0.001) but no statistical difference in average eating behavior score.

**Conclusion**: The study found that the use of mobile health education to deliver health programs facilitates communication between the healthcare provider and individual, and can empower adolescent females in their pursuit of weight loss by improving their attitudes and knowledge, leading to better health behavior.

## Introduction

Being overweight or obese is known to be an important factor in many serious health issues, and approximately 10% of global health expenditure is spent on fighting obesity. ^1, 2^ Nearly one-third of Thai university students are reported to be overweight, and Thailand was ranked second highest for obesity in the Southeast Asia region in 2013. ^3^ Studies have proposed face-to-face consultation interventions for weight loss among obese adolescents across the globe based on the health belief model (HBM) and social support theories in different parts of the globe. ^4, 5^ Such intervention requires significant funding and, over the last decade, the Thai government spent around five billion Baht on the health problem caused by obesity ^6^; face-to-face activity spent more than 50% of their budget for food expenditure. Two particularly effective programs aimed at controlling obesity involved sending informative health education messages to smartphones. ^7, 8^ The younger section of the population, especially students, regularly use smartphones to interact on social networks, such as Facebook. ^9– 11^ It has been demonstrated that using social networks can be a useful approach in research and can strengthen disease prevention interventions and health promotion programs for control of weight and obesity. ^12^ Mobile health education (MHE) is a simple means of supporting interactions with an individual and represents a cost-effective intervention approach. Thus, MHE has been used as tool to promote better health. ^13, 14^ The HBM states that the perception of a personal health behavior influences the consequences of a particular health problem. ^15^ The HBM has been used to explain and predict preventative health behavior that can influence an individual’s decision making, which can be measured. ^16^ Social support networks are present in relationships involving healthcare behaviors, especially among groups of women. It has been shown that women are more satisfied if their networks are wide, whereas men are more satisfied if their networks are small. ^17^

MHE could positively influence an individual's behavior based on HBM and social support theory. Therefore, this study used MHE to deliver a weight management program to adolescent females in Thailand and compared this method with a face-to-face health education (FHE) activity.

## Methods

### Ethical statement

Written informed consent was obtained from participants prior to the start of the study. Ethical approval was granted by the Ethics Review Committee for Research Involving Human Subjects of Chulalongkorn University, Thailand (COA No. 142.1/60).

### Study design

This was a comparative cross-sectional pre–post study with three groups: face-to-face consultation (FHE), mobile health education message (MHE) and an observation (control) group.

### Sample size and selection

**Figure 1.**
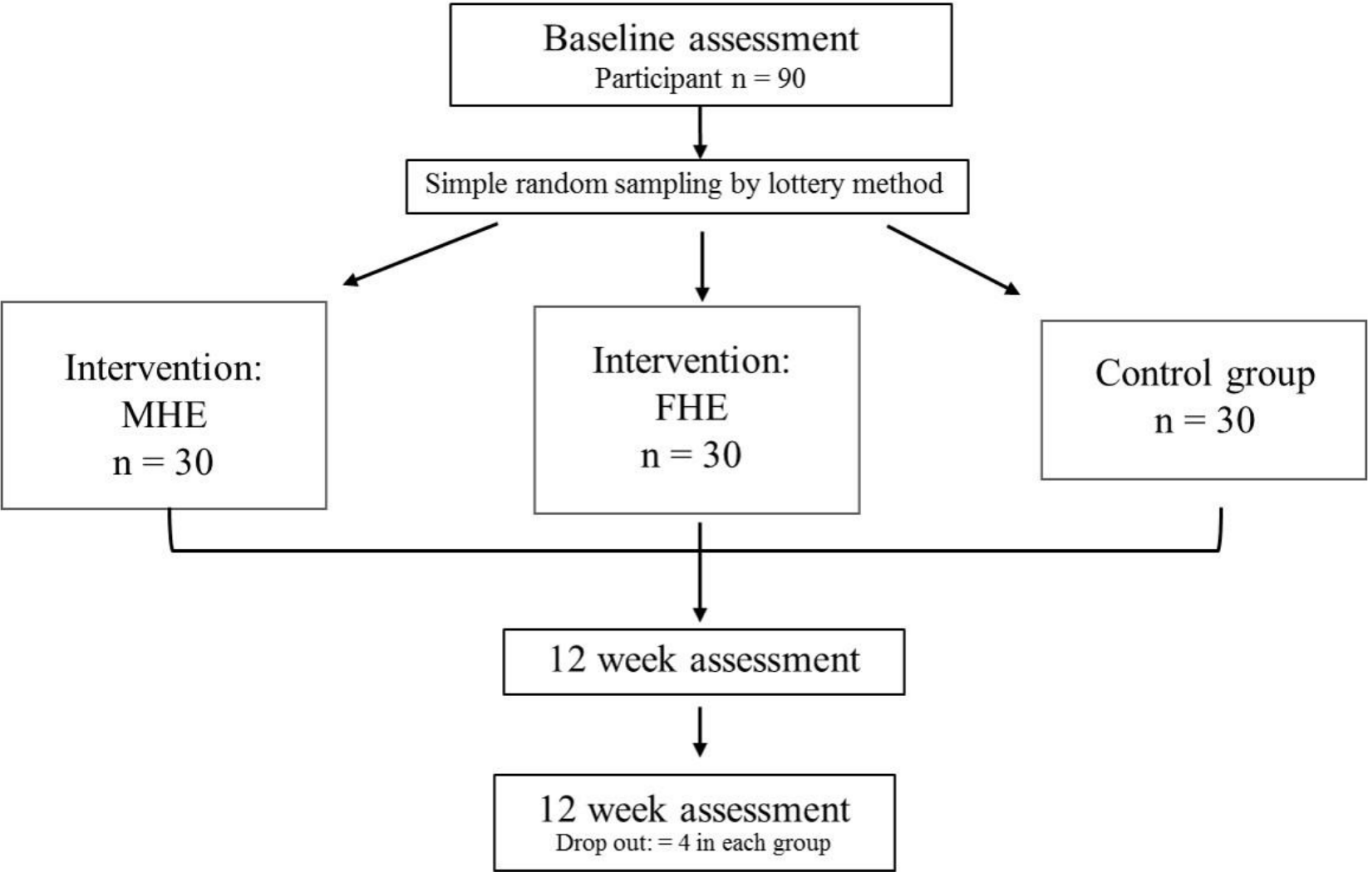
Study flow diagram.

Participants were invited through open advertisement on faculty boards at Srinakharinwirot University, Thailand and were screened according to the inclusion and exclusion criteria. Participants in the two test groups received a health education in the form of a weight management program either via Facebook or through face-to-face consultation; the control group received neither and was only observed ( Figure 1). A sample size of 90 (including 20% drop out) was calculated with G*Power, ^18^ using a power of 0.80, alpha value of 0.05, and a correlation between pre- and post-intervention of 0.5 was assumed. Each group was allocated 30 participants through simple random method by allocating an equal number in each group. Four participants from each group were unable to complete the end line assessment due to their personnel reasons. However; the response rate was 87% in this study. Inclusion criteria was the faculty members from one of three faculties at Srinakharinwirot University with BMI ≥ 23 and < 25; and for the Facebook group only they also had to have access to Facebook at least once per day. Those were excluded from the study who were disabled, physically inactive, had an underlying disease that could cause abnormal weight loss or gain; or (3) had participated in any other weight management program/trial within the previous six months.

**Data collection**

The dependent variables of this study were weight, body mass index (BMI, calculated as the weight [kilograms-kg] of a participant divided by the square of their height [meters-m]), waist–hip ratio (WHR, measured at the midpoint between the lower margin of the least palpable rib and the top of the iliac crest, using a stretch resistant tape that provided a constant 100 g tension), health beliefs, social support, and health behavior (eating and physical activity). Health beliefs were measured by using the HBM questionnaire, ^19^ which contains six modules, and social support was measured with a questionnaire ^20^ containing two modules. Physical activity was assessed by using the Global Physical Activity Questionnaire (GPAQ) developed by the World Health Organization (WHO) which comprised three domains: activity at work, travel to and from places, and recreational activities. The results describing participants’ physical activity were expressed as metabolic equivalents (METs), which reflected the intensity of physical activities as MET-minutes per day, ^21^ and eating behavior was recorded through a self-evaluation questionnaire on eating behavior developed by the Department of Health (Thailand). ^22^

The weight management program focused on weight loss. It presented information via an infographic message because it was easier to understand and recognize. ^23^ The information provided was about being overweight, diet and exercise for weight loss, the severity of obesity, and the benefits of losing weight as described by the Thai Health Promotion Foundation. ^24^ In the MHE group, infographics were posted to Facebook once a day, whereas question and answer activities took place biweekly via private messaging in Facebook for 12 weeks. In the FHE group, participants received a health information booklet and took part in biweekly group activity that included an individual test and an interactive discussion, for 12 weeks. The third group (control) was just observed and compared with the MHE and FHE groups at the end of 12 weeks and again at the total 24 week study period by all assessment (weight, BMI, WHR and questionnaire).

**Statistical analysis**

Two-way repeated measures ANOVA tests and Fisher’s least significant difference (LSD) post hoc tests were used to analyze the data. Data was expressed as mean ± SD; statistical significance was considered at *p* < 0.05.

## Results

The average age of participants was 18.23 ± 0.42 years in the control group, 18.17 ± 0.37 years in the MHE group, and 18.20 ± 0.4 years in the FHE group. The baseline values for all variables, including weight, BMI, WHR, the six HBM modules, two social support modules, and two health behavior modules, are summarized in Table 1.

**Table 1.** Basic information in each group at baseline ( *n* = 78).

Variable	Participant group			*p* value
	Control group mean (SD) *n* = 26	MHE (Facebook) mean (SD) *n* = 26	FHE (face-to-face) mean (SD) *n* = 26	
Age [years]	18.23 (0.42)	18.17 (0.37)	18.20 (0.4)	0.81
Weight [kg]	62.9 (4.27)	61.8 (4.6)	64.3 (4.0)	0.142
BMI [kg/m ^2^]	23.8 (0.86)	23.6 (0.75)	24 (0.77)	0.16
WHR	0.84 (0.04)	0.83 (0.03)	0.82 (0.02)	0.051
HBM 1	3.13 (0.39)	3.07 (0.48)	3.2 (0.27)	0.51
HBM 2	3.47 (0.39)	3.25 (0.40)	3.57 (0.45)	0.11
HBM 3	2.66 (0.45)	2.64 (0.37)	2.74 (0.50)	0.48
HBM 4	2.76 (0.41)	2.70 (0.29)	2.83 (0.61)	0.57
HBM 5 (eat)	2.90 (0.29)	2.92 (0.31)	2.85 (0.27)	0.77
HBM 5 (ex)	2.82 (0.28)	2.76 (0.23)	2.89 (0.41)	0.26
Social support (eat)	2.74 (0.38)	3.13 (0.54)	2.59(0.80)	0.08
Social support (ex)	2.82 (0.51)	2.77 (0.43)	3.11(0.74)	0.07
Eating behavior	40.86 (0.28)	39.73 (0.30)	39.81 (0.29)	0.1
MET	580 (35.9)	547.38 (38.65)	523.70 (37.93)	0.54

After the 12 weeks intervention period, all variables were tested for interaction between group and time. When a variable showed p < 0.05 then that variable had to test pairwise as described in Table 2. The results from the two-way repeated ANOVA test showed statistically significant differences between the intervention and control groups, within group and the interaction effect taken at the start and end of the study.

**Table 2.** Repeated measure ANOVA of all measurement variable.

Source measure		*SS*	*df*	*MS*	*F*	*p* value
**Weight**	Time × group	13.48	2.85	4.72	12.85	<0.001 *
	group	319.77	2	159.89	3.04	0.054
**BMI**	Time × group	1.68	3.54	0.47	4.62	< 0.01 *
	group	16.18	2	8.09	4.46	0.015 *
**WHR**	Time × group	1.19	2.46	4.87	0.49	0.654
	group	0.21	2	0.1	3.15	0.049 *
**HBM1**	Time × group	1.79	3.23	0.54	6.08	<0.001 *
	group	3.30	2	1.65	9.53	<0.001 *
**HBM2**	Time × group	32.68	2.78	11.73	116.71	<0.001 *
	group	6.17	2	3.08	12.69	<0.001 *
**HBM3**	Time × group	7.07	3.44	2.05	15.73	<0.001 *
	group	9.46	2	4.72	21.51	<0.001 *
**HBM4**	Time × group	4.57	3.41	1.34	10.75	<0.001 *
	group	9.31	2	4.66	17.21	<0.001 *
**HBM5 (eat)**	Time × group	2.46	3.38	0.73	15.68	<0.001 *
	group	4.3	2	2.15	17.32	<0.001 *
**HBM5 (ex)**	Time × group	2.59	3.06	0.72	10.06	<0.001 *
	group	4.58	2	2.29	15.47	<0.001 *
**Social support (eating)**	Time × group	4.25	4	1.06	6.82	<0.001 *
	group	26.24	2	13.12	24.55	<0.001 *
**Social support (exercise)**	Time × group	13.69	3.45	3.96	19.13	<0.001 *
	group	21.57	2	10.78	18.52	<0.001 *
**Health behavior (eating)**	Time × group	40.03	3.61	11.09	7.68	>0.001 *
	group	10.95	2	5.47	1.904	0.156
**Health behavior (physical activity: MET)**	Time × group	1382855.45	3.28	421969.46	12.60	>0.001 *
	group	1296386.70	2	648193.35	8.434	>0.001 *

* The mean difference is significant at the 0.05 level.

### Comparisons between groups

According to the analysis, there were no significant differences between groups for weight and eating behavior. Therefore, pairwise testing was conducted for all other variables (BMI, WHR, all HBM modules, both social support modules, and MET physical activity; Table 3).

**Table 3.** Pairwise comparisons results; MHE ( *n* = 26), FHE ( *n* = 26) and control groups ( *n* = 26).

Variable	Group pairing (i−j)	Mean difference (i−j)	*p* value
BMI	Control–MHE Control–FHE MHE–FHE	0.55 −0.01 −0.56	0.013 * 0.953 0.011 *
WHR	Control–MHE Control–FHE MHE–FHE	0.02 0.03 −0.07	0.016 * 0.098 0.437
HBM 1	Control–MHE Control–FHE MHE–FHE	−0.204 −0.282 −0.078	0.003 * <0.001 * 0.244
HBM 2	Control–MHE Control–FHE MHE–FHE	0.029 0.358 0.329	0.710 <0.001 * <0.001 *
HBM 3	Control–MHE Control–FHE MHE–FHE	0.425 0.427 0.001	<0.001 * <0.001 * 0.988
HBM 4	Control–MHE Control–FHE MHE–FHE	−0.389 −0.451 −0.062	<0.001 * <0.001 * 0.459
HBM 5 (eat)	Control–MHE Control–FHE MHE–FHE	−0.288 −0.287 0.001	<0.001 * <0.001 * 0.989
HBM 5 (ex)	Control–MHE Control–FHE MHE–FHE	−0.250 −0.328 −0.078	<0.001 * <0.001 * 0.207
Social support (eat)	Control–MHE Control–FHE MHE–FHE	−0.738 −0.109 0.629	<0.001 * 0.336 0.000 *
Social support (ex)	Control–MHE Control–FHE MHE–FHE	−0.577 −0.646 −0.069	<0.001 * <0.001 * 0.558
MET	Control–MHE Control–FHE MHE–FHE	−175.15 −131.41 43.74	<0.001 * 0.004 * 0.328

* The mean difference is significant at the 0.05 level.

From the pairwise group analysis, the mean BMI and WHR in the MHE group were significantly lower than the control group. There was no significant difference in weight change between groups, but BMI and WHR showed significant differences in each group. The pairwise results revealed that the BMI of the control group was significantly higher than that of the MHE group but lower than the FHE group with no statistically significance. The average BMI of the MHE group was significantly lower than the average BMI of the FHE group. Whereas the average WHR of the control group was significantly higher than the MHE group and also higher than the FHE group, no significant difference was found. Moreover, the average WHR of the FHE group was higher than the MHE group but not significance.

The pairwise analysis of the HBM modules showed significantly different average scores between the control group and both intervention groups, with the exception of HBM 2 (perceived benefits of weight loss), which showed no significant difference between the control and MHE groups but did show a significant difference between the MHE and FHE groups ( *p* < 0.001). The average scores for the HBM 1, HBM 3, HBM 4, HBM 5 (eat) and HBM 5 (ex) modules of the MHE and FHE groups were significantly higher than those of the control group. However, the average scores on the HBM 1, HBM 4 and HBM 5 (ex) modules for the FHE group were higher than those of the MHE group, but not statistically significantly so. Meanwhile, the average scores on the HBM 2, modules for the MHE group was significantly higher than those of the FHE group.

The average scores of social support exercise modules of the intervention groups were significantly higher than the average score from the control group. While the average score of social support eating module showed significance higher than control group only in MHH group. The MHE group showed a significantly higher score of social support (eating behavior) than the FHE group. Whereas the social support (exercise behavior) score of the FHE group was higher than that of the MHE group, it was not statistically different. The pairwise analysis between groups revealed that the average MET scores of both intervention groups were significantly higher than that of the control group. The MHE group averaged a higher MET score than the FHE group, but this difference was not statistically significant.

When the differences between time points were analyzed, WHR was the only variable that was not statistically significantly different across the study period. Therefore, pairwise testing was conducted for all other variables (weight, BMI, all HBM modules, both social support modules and both health behavior modules; Table 4).

**Table 4.** Pairwise comparisons between the different time periods for baseline, 12 weeks (after intervention), and 24 weeks (after follow-up).

Variable	Time pair (i−j)	Mean difference (i−j)	*p* value
Weight	Baseline–12 weeks Baseline–24 weeks 12 weeks–24 weeks	0.436 0.442 0.006	<0.001* <0.001* 0.903
BMI	Baseline–12 weeks Baseline–24 weeks 12 weeks–24 weeks	0.231 0.154 −0.077	<0.001* 0.007* 0.058
HBM 1	Baseline–12 weeks Baseline–24 weeks 12 weeks–24 weeks	−0.242 −0.239 0.003	<0.001* <0.001* 0.934
HBM 2	Baseline–12 weeks Baseline–24 weeks 12 weeks–24 weeks	−0.068 0.548 0.616	<0.001* 0.009* <0.001*
HBM 3	Baseline–12 weeks Baseline–24 weeks 12 weeks–24 weeks	0.344 0.347 0.003	<0.001* <0.001* 0.947
HBM 4	Baseline–12 weeks Baseline–24 weeks 12 weeks–24 weeks	−0.049 −0.044 0.045	<0.001* <0.001* 0.361
HBM 5 (eat)	Baseline–12 weeks Baseline–24 weeks 12 weeks–24 weeks	−0.037 −0.338 0.028	<0.001* <0.001* 0.334
HBM 5 (ex)	Baseline–12 weeks Baseline–24 weeks 12 weeks–24 weeks	−0.254 −0.209 0.046	<0.001* <0.001* 0.185
Social support (eat)	Baseline–12 weeks Baseline–24 weeks 12 weeks–24 weeks	−0.446 −0.259 0.187	<0.001* <0.001* 0.002*
Social support (ex)	Baseline–12 weeks Baseline–24 weeks 12 weeks–24 weeks	−0.605 −0.303 0.303	<0.001* <0.001* <0.001*
Eating behavior	Baseline–12 weeks Baseline–24 weeks 12 weeks–24 weeks	−1.128 −0.487 0.641	<0.001* 0.021* 0.182
MET	Baseline–12 weeks Baseline–24 weeks 12 weeks–24 weeks	−217.282 −51.513 165.769	<0.001* 0.039* <0.001*

When comparing the results at different points in the study, the analysis showed that average weight and BMI measured at baseline were significantly higher than at post-intervention and follow-up, but there were no significant differences between post-intervention and follow-up. There were no statistically significant differences in average WHR between baseline measurements and either post-intervention or follow-up.

A comparison of the HBM scores at different time points revealed that the baseline of all HBM modules differed significantly between the intervention period and the follow-up period ( *p* < 0.001). However, there was no significant difference between the end of the intervention period and the follow-up period, except for HBM 2.

The pairwise analysis of the three study phases (baseline, post-intervention and follow-up) showed that the average scores of HBM 1, HBM 4, HBM 5 (eat), and HBM 5 (ex) measured at baseline were significantly lower than those measured post-intervention and at follow-up. Although the average score of the HBM 2 module measured at baseline was significantly lower than the score post-intervention, it was significantly higher than the score at follow-up. The average score of HBM 3 at baseline was significantly higher than post-intervention and at follow-up. HBM 2 was only HBM module for which the post-intervention score was significantly higher than at follow-up, whereas the other HBM modules showed no significant differences between post-intervention and follow-up.

A pairwise comparison of social support at the different study points showed that the average scores for social support for eating and exercise at baseline were significantly lower than at post-intervention or follow-up ( *p* < 0.001). In addition, the results of both social support modules showed that the average scores post-intervention were significantly higher than at follow-up. The pairwise comparison of study points revealed that the average MET and average eating behavior scores at baseline were significantly lower than post-intervention or at follow-up. Moreover, the average MET and average eating behavior scores measured post-intervention were higher than at follow-up. There was a statistical difference in average MET ( *p* = 0.039) but no statistical difference in average eating behavior score.

## Discussion

As expected, the baseline characteristics among the three groups in this study were similar. The average weight of both intervention groups decreased after both the initial period and at follow-up. This is consistent with the findings of previous studies, which found that social media can encourage people to lose weight through social interactions on online notice boards or forums. ^25, 26^ Likewise, BMIs at post-intervention and follow-up were significantly lower than at baseline, a finding consistent with previous studies. ^27, 28^ Pairwise comparisons of the three groups revealed that the MHE group had the highest average scores for perceived benefits, barriers, and self-efficacy in dietary life. The FHE group showed the highest score in perceived threat, cues to action, and self-efficacy in exercise. These findings are consistent with those of a previous study, which found that print or electronic media (an external cue) impacted BMI through the HBM (specifically, perceived benefit, perceived barriers, and perceived threat or severity). ^29^

The FHE group had the highest average score for the perceived threat of being overweight, cues to action for weight loss, and perceived self-efficacy in exercise. These are modules that require motivation to participate in activities such as face-to-face meetings (e.g., counseling) or planned exercise with another person. ^30^ In addition, the perceived threats for adolescents, is about acceptance by their peers, and the participants expressed higher satisfaction if they participated in activities with friends. Previous studies found that female high-school and college students who had a role within their team changed their behavior when they perceived a threat or severity. ^29, 31^

Social support was consistent with the results of the HBM in the self-efficacy module; the MHE group had a lower score in the exercise module but a higher score for diet behavior. There was a significant difference in physical activity between groups because behavior modification is different from awareness or belief. Although awareness occurred, behavior might not have changed. This is consistent with previous research that found that online social support had a much greater influence on eating behavior than for exercise behavior. ^32^

However, the results revealed that both intervention groups showed significantly higher average MET scores than the control group. When considering the interaction between the time point of HBM, social support, and health behavior, most of the variables had similar average scores post-intervention, which were higher than baseline and at follow-up. This corresponds well to the findings of previous studies that found that intervention via EHE serves as information support, which is needed to be a successful weight loss program. ^32^ In addition, a previous study revealed that participants who took part in a campaign of physical activity based on self-efficacy via EHE showed significant increasing medium MET scores compared with other groups that received printed brochures. ^33^ The findings of another study revealed that motivation decreased when intervention ceased, resulting in reduced friend support and confidence to action because participants lack the ability to share and be supported by friends. ^34^

### Limitations

Some participants were still in growing up age; therefore, their height has an effect on BMI which could be stable or decrease despite of no change in weight and health behavior. Besides, the intervention has short-term effects because the follow up showed a trend of decreasing health belief, social support, and health behavior but showed an increasing trend in anthropometric assessment. Hence this study cannot be generalized in the whole country due to the limited nature of the population involved.

## Conclusions

The data suggests that mobile health education is an effective approach for improving behavior towards weight reduction among obese adolescent females studying at Thai universities. Especially, in a condition where all activities must be based on principles of social distancing, using online social network sites to access activities or programs can stimulate feelings by inducing personal satisfaction. In addition, the program or information provided online should encourage participants to participate frequently in order to promote social support similar to what they receive from face-to-face activities.

## Data availability

### Underlying data

Open Science Framework. Comparison of mobile health education messages verses face-to-face consultation for weight reduction among overweight female adolescents in Thailand. OSF 2021. https://doi.org/10.17605/OSF.IO/4KPJ9. ^35^

This project contains the following underlying data:

• Source data for all table. (Raw data of each table)
• Raw data Supim.xlsx (Deidentified raw data in excel file pattern)
• Raw data Supim.sav (Deidentified raw data in SPSS file pattern)

### Extended data

Open Science Framework. Comparison of mobile health education messages verses face-to-face consultation for weight reduction among overweight female adolescents in Thailand. OSF 2021. https://doi.org/10.17605/OSF.IO/4KPJ9. ^35^

This project contains the following extended data:

• Questionnaire Supim.pdf. (the questionnaire used in this study).

Data are available under the terms of the Creative Commons Zero “No rights reserved” data waiver (CC0 1.0 Public domain dedication).

## Reporting guidelines

Open Science Framework. STROBE checklist for ‘Comparison of mobile health education messages verses face-to-face consultation for weight reduction among overweight female adolescents in Thailand’. https://doi.org/10.17605/OSF.IO/4KPJ9. ^35^

Data are available under the terms of the Creative Commons Zero “No rights reserved” data waiver (CC0 1.0 Public domain dedication).
