# Supplementary material for: Comparison of mobile health education messages verses face-to-face consultation for weight reduction among overweight female adolescents in Thailand
Source: F1000Res. 2022 Nov 21;10:635. Originally published 2021 Jul 22. [Version 2] doi: 10.12688/f1000research.51156.2 (PMC9727315; doi:10.12688/f1000research.51156.2)
Supplement: Peer review history

# Peer review: Reviewer response for version 2

**DOI:** 10.5256/f1000research.140230.r156235

**Published:** 2022-12-06

**Authors:** Umer Farooq

**Affiliations:** 1 Community medicine Department, Ayub medical College, Abbottabad, Pakistan

I have gone through the revisons in the article and the response of the author to the comments and found it satisfactory.

Is the work clearly and accurately presented and does it cite the current literature?

No

If applicable, is the statistical analysis and its interpretation appropriate?

Yes

Are all the source data underlying the results available to ensure full reproducibility?

Yes

Is the study design appropriate and is the work technically sound?

No

Are the conclusions drawn adequately supported by the results?

Yes

Are sufficient details of methods and analysis provided to allow replication by others?

Yes

Reviewer Expertise:

Epidemiology, Health Statistics, Public health

I confirm that I have read this submission and believe that I have an appropriate level of expertise to confirm that it is of an acceptable scientific standard.

# Peer review: Reviewer response for version 2

**DOI:** 10.5256/f1000research.140230.r156234

**Published:** 2022-11-28

**Authors:** Kulnipa Kittisakmontri

**Affiliations:** 1 Division of Nutrition, Department of Pediatrics, Faculty of Medicine, Chiang Mai University, Chiang Mai, Thailand

I have no further comments.

Is the work clearly and accurately presented and does it cite the current literature?

Partly

If applicable, is the statistical analysis and its interpretation appropriate?

Partly

Are all the source data underlying the results available to ensure full reproducibility?

Yes

Is the study design appropriate and is the work technically sound?

No

Are the conclusions drawn adequately supported by the results?

Yes

Are sufficient details of methods and analysis provided to allow replication by others?

No

Reviewer Expertise:

Pediatric nutrition

I confirm that I have read this submission and believe that I have an appropriate level of expertise to confirm that it is of an acceptable scientific standard.

# Peer review: Reviewer response for version 1

**DOI:** 10.5256/f1000research.54287.r150532

**Published:** 2022-09-26

**Authors:** Kulnipa Kittisakmontri

**Affiliations:** 1 Division of Nutrition, Department of Pediatrics, Faculty of Medicine, Chiang Mai University, Chiang Mai, Thailand

The findings of this study are interesting, but the manuscript need major revision to meet the standard requirement of scientific research. 

**Substantial considerations:**

 1) Study design

- It was unusual to call a cross-sectional study in case that some interventions were given to the study population.
- I wonder whether simple randomization by lottery method adequately allocated participants in each groups? If the answer is yes, why did the authors called the study RCT? It was very confusing when the manuscript mentioned this study as the 'cross-sectional study' but the flow chart showed that participants were allocated in each group by simple randomization.

2) Sample size calculation

- I didn't understand the method used to determine sample size. What did the correlation pre- and post mentioned by authors for example, correlation of body weight or BMI or behavioral scores? More importantly, it needed a reference for the number used in calculation - where did the correlation of 0.5 come from? It was very unclear to me how this formula was used to determine sample size could relate to main research questions of this study.

3) Study population

- The inclusion criteria were unclear. Which were the three faculties that authors had mentioned? Was there any selection bias if the authors had selected the MHE from a population who frequently used the Facebook than others? It might be assumed that the frequent users of Facebook would use variety of Social media platforms that could expose them to more knowledge of weight loss information or motivation from famous actress or models they admired. At least this issue should be discussed as a limitation of the study.

4) Methodology

- The authors should describe more details of the interventions and questionnaires used in this study. The validation of those questionnaires should be mentioned to confirm their accuracy and reproducibility. 

5) Results

- In general, I suggest the authors to seek advices from the medical statistician for the presentation of research findings. I was very confuse with the presentation of the results. All tables should stand alone with clear messages. For those figures and tables, the titles and all abbreviations should be clarified. I suggest the authors should extensively revise all figure and tables. Moreover, the reported numbers should be consistent with specific decimal. If two decimal was used, the authors should write all reported number in two decimal.
- Table 2 - it was very unclear to me what the authors would like to present. The authors should clarify what time point that those results came from (at post intervention?). Basically, I think table 2 and 3 could be integrated into one table when the authors showed the results of all variables divided by groups and showed the post hoc p-value of each pairs. If the post hoc analysis show significant results, thus it can assume the significant p-value of the repeated ANOVA.
- Table 4 - I didn't know the population of this analysis whether only intervention groups were counted or all participants were included. 

6) Discussion

- The discussion section should be intensively organized. I found some repetition of some sentences mentioning the same statement. Furthermore, there were many limitations that should be mentioned, for example, as I said before the selection bias of the MHE group, the nature of the study especially in case the authors were not sure with their random allocation, thus the authors cannot infer the causality between the interventions and outcomes because the study was not the RCT. 

7) Abstract

- The sentences mentioning that 'the group received MHE had the lowest BMI and WHR' shouldn't be included in the abstract as these could lead to misunderstanding that the MHE groups clearly had benefit from the intervention while the results presented in the manuscript did not demonstrate that.

**Minor concerns:**

- Please check repetition of the sentences and typos carefully. 

Is the work clearly and accurately presented and does it cite the current literature?

Partly

If applicable, is the statistical analysis and its interpretation appropriate?

Partly

Are all the source data underlying the results available to ensure full reproducibility?

Yes

Is the study design appropriate and is the work technically sound?

No

Are the conclusions drawn adequately supported by the results?

Yes

Are sufficient details of methods and analysis provided to allow replication by others?

No

Reviewer Expertise:

Pediatric nutrition

I confirm that I have read this submission and believe that I have an appropriate level of expertise to state that I do not consider it to be of an acceptable scientific standard, for reasons outlined above.

# Response

**Published:** 2022-10-24

**Authors:** supim Wongtongtair

**Affiliations:** Srinakharinwirot University, Thailand

Study design

- It was unusual to call a cross-sectional study in case that some interventions were given to the study population.
- I wonder whether simple randomization by lottery method adequately allocated participants in each groups? If the answer is yes, why did the authors called the study RCT? It was very confusing when the manuscript mentioned this study as the 'cross-sectional study' but the flow chart showed that participants were allocated in each group by simple randomization.

***Response***: This was Quasi-experimental study design, We have corrected this mistake.

 2) Sample size calculation

- I didn't understand the method used to determine sample size. What did the correlation pre- and post mentioned by authors for example, correlation of body weight or BMI or behavioral scores? More importantly, it needed a reference for the number used in calculation - where did the correlation of 0.5 come from? It was very unclear to me how this formula was used to determine sample size could relate to main research questions of this study.

***Response***: We assumed 50% difference after the intervention, as we were unable to find out the exact difference in score in other interventional studies. Moreover, we have revised this sentence for more clarity.

 3) Study population

- The inclusion criteria were unclear. Which were the three faculties that authors had mentioned? Was there any selection bias if the authors had selected the MHE from a population who frequently used the Facebook than others? It might be assumed that the frequent users of Facebook would use variety of Social media platforms that could expose them to more knowledge of weight loss information or motivation from famous actress or models they admired. At least this issue should be discussed as a limitation of the study.

***Response***: We have included this in the limitation as per the guidance.

 4) Methodology

- The authors should describe more details of the interventions and questionnaires used in this study. The validation of those questionnaires should be mentioned to confirm their accuracy and reproducibility.

***Response***: We have added this information in method section as per the guidance. 

 5) Results

- In general, I suggest the authors to seek advices from the medical statistician for the presentation of research findings. I was very confuse with the presentation of the results. All tables should stand alone with clear messages. For those figures and tables, the titles and all abbreviations should be clarified. I suggest the authors should extensively revise all figure and tables. Moreover, the reported numbers should be consistent with specific decimal. If two decimal was used, the authors should write all reported number in two decimal.

***Response***: We have corrected as per the guidance

- Table 2 - it was very unclear to me what the authors would like to present. The authors should clarify what time point that those results came from (at post intervention?). Basically, I think table 2 and 3 could be integrated into one table when the authors showed the results of all variables divided by groups and showed the post hoc p-value of each pairs. If the post hoc analysis show significant results, thus it can assume the significant p-value of the repeated ANOVA.

***Response***: We have merged the table 2 as per the guidance.

- Table 4 - I didn't know the population of this analysis whether only intervention groups were counted or all participants were included. 

***Response*** **:** We have included final 26 participants in the analysis. 4 from each group who did not complete the intervention were excluded in the analysis.

 6) Discussion

- The discussion section should be intensively organized. I found some repetition of some sentences mentioning the same statement. Furthermore, there were many limitations that should be mentioned, for example, as I said before the selection bias of the MHE group, the nature of the study especially in case the authors were not sure with their random allocation, thus the authors cannot infer the causality between the interventions and outcomes because the study was not the RCT. 

***Response***: Agreed and revised as per the suggestions.

 7) Abstract

- The sentences mentioning that 'the group received MHE had the lowest BMI and WHR' shouldn't be included in the abstract as these could lead to misunderstanding that the MHE groups clearly had benefit from the intervention while the results presented in the manuscript did not demonstrate that.

***Response***: Revised as per kind guidance.

**Minor concerns:**

- Please check repetition of the sentences and typos carefully.

# Peer review: Reviewer response for version 1

**DOI:** 10.5256/f1000research.54287.r90391

**Published:** 2021-08-18

**Authors:** Umer Farooq

**Affiliations:** 1 Community medicine Department, Ayub medical College, Abbottabad, Pakistan

- The study covers an important topic and provides evidence based results regarding an important intervention. 
- Most of the references used are more than 6 years old. The author may look for some more studies recently done and include them in the write up.
- This is a cross sectional study. Why it was not planned as an experimental study? The researchers are doing the intervention then why wasn't it planned as an experimental study?
- The researchers have done a good analysis and have explained the results well as of a cross sectional study.
- The external validity of the study is very limited as it applies only to the girls of that university.

Is the work clearly and accurately presented and does it cite the current literature?

No

If applicable, is the statistical analysis and its interpretation appropriate?

Yes

Are all the source data underlying the results available to ensure full reproducibility?

Yes

Is the study design appropriate and is the work technically sound?

No

Are the conclusions drawn adequately supported by the results?

Yes

Are sufficient details of methods and analysis provided to allow replication by others?

Yes

Reviewer Expertise:

Epidemiology,Health Statistics,Public health

I confirm that I have read this submission and believe that I have an appropriate level of expertise to confirm that it is of an acceptable scientific standard, however I have significant reservations, as outlined above.

# Response

**Published:** 2022-04-20

**Authors:** supim Wongtongtair

**Affiliations:** Srinakharinwirot University, Thailand

**Reviewer:** The study covers an important topic and provides evidence based results regarding an important intervention.

**Response:** Thank you for understanding our efforts.

**Reviewer:** Most of the references used are more than 6 years old. The author may look for some more studies recently done and include them in the write up.

**Response:** I agree for this and adding some recently study already but can only change some references because this work was done in 2018. Therefore some data which used as rational or theory of the study cannot changed. However, after this editing reference more than 75% will be in period of 2017-2021.

**Reviewer:** This is a cross sectional study. Why it was not planned as an experimental study? The researchers are doing the intervention then why wasn't it planned as an experimental study?

**Response:** This issue is in line with our concern too. Because 3 groups of this study was done in same university. Therefore we try to decrease contamination by specify faculty which less likely to encounter each other. However, we choose faculties where students have similar academic and social characteristics. For the reasons mentioned above, we therefore use the cross sectional design with pre –post study.

**Reviewer:** The researchers have done a good analysis and have explained the results well as of a cross sectional study.

**Response:** Thank you for the compliment.

**Reviewer:** The external validity of the study is very limited as it applies only to the girls of that university.

**Response:** It's an issue that we used to be afraid of. However, this study is based on HBM and social support principle which the universal theory to use for weight management in adolescent. Moreover, our results were also consistent with similar previous research as you seen in discussion.

# Response

**Published:** 2022-06-29

**Authors:** supim Wongtongtair

**Affiliations:** Srinakharinwirot University, Thailand

**Reviewer:** The study covers an important topic and provides evidence based results regarding an important intervention.

**Response:** Thank you for understanding our efforts.

**Reviewer:** Most of the references used are more than 6 years old. The author may look for some more studies recently done and include them in the write up.

**Response:** I agree for this and adding some recently study already but can only change some references because this work was done in 2018. Therefore some data which used as rational or theory of the study cannot changed. However, after this editing reference more than 75% will be in period of 2017-2021.

**Reviewer:** This is a cross sectional study. Why it was not planned as an experimental study? The researchers are doing the intervention then why wasn't it planned as an experimental study?

**Response:** This issue is in line with our concern too. Because 3 groups of this study was done in same university. Therefore we try to decrease contamination by specify faculty which less likely to encounter each other. However, we choose faculties where students have similar academic and social characteristics. For the reasons mentioned above, we therefore use the cross sectional design with pre–post study.

**Reviewer:** The researchers have done a good analysis and have explained the results well as of a cross sectional study.

**Response:** Thank you for the compliment.

**Reviewer:** The external validity of the study is very limited as it applies only to the girls of that university.

**Response:** It's an issue that we used to be afraid of. However, this study is based on HBM and social support principle which the universal theory to use for weight management in adolescent. Moreover, our results were also consistent with similar previous research as you seen in discussion.
